# CHASERR-CHD2 dynamics in T cell quiescence and its modulation by cyclosporine

**DOI:** 10.3389/fimmu.2025.1652359

**Published:** 2025-11-11

**Authors:** Anna Budkina, Anatoliy Zubritskiy, Daria Marakulina, Marina Yu. Loguinova, Nikita A. Sergeev, Yulia A. Medvedeva

**Affiliations:** 1 Institute of Bioengineering, Research Center of Biotechnology Russian Academy of Science, Moscow, Russia; 2 Moscow Center for Advanced Studies, Moscow, Russia; 3 Flow Cytometry Group, Endocrinology Research Centre, Moscow, Russia

**Keywords:** lncRNA, transcription, single-cell, T cell activation, T cell quiescence

## Abstract

**Background:**

CHASERR, a conserved long non-coding RNA located upstream of CHD2, transcriptionally represses CHD2 in *cis*. Both genes are highly expressed in lymphocytes, suggesting roles in immune regulation, though their functions remain undefined.

**Results:**

We identified elevated expression of CHASERR and CHD2 in naïve and regulatory T cells through analysis of single-cell and bulk RNA-seq datasets. Both their promoters are bound by FOXP3, the key regulator of Treg cells, and FOXP1, the key regulator of naïve T cell quiescence. Expression dynamics during early T cell activation revealed that a decline in CHASERR precedes a transient increase in CHD2. Correlation analysis linked CHASERR/CHD2 expression to quiescence-associated genes, suggesting a role in maintaining T cell homeostasis. We predicted and experimentally validated that cyclosporine A, a calcineurin inhibitor and potent immunosuppressant, mitigates the transcriptional changes induced by CHASERR loss, notably reducing elevated CHD2 expression *in vitro* after CHASERR knockdown.

**Conclusions:**

Our results position the CHASERR-CHD2 axis as a potential regulator of T cell homeostasis and activation. Furthermore, we propose cyclosporine A as a potential therapeutic strategy for conditions involving CHASERR deficiency.

## 1 Introduction

Long noncoding RNAs (lncRNAs) have been associated with different immune cell functions, such as cell differentiation, activation, cell migration, and cytokine production (1). By interacting with transcription factors and chromatin-modifying proteins, lncRNAs function as important regulators of the expression of genes associated with inflammation (2). Recent research has shown the involvement of lncRNA regulators in various inflammatory and autoimmune diseases (3, 4).

CHASERR is a conserved lncRNA located upstream of the protein-coding gene CHD2 on the same strand. Rom et al. (5) have demonstrated that the loss of CHASERR leads to an increase in CHD2 mRNA and protein levels and that CHASERR acts *in cis* to repress CHD2 expression. While the precise molecular mechanism underlying this repression remains incompletely understood, current hypotheses suggest that the repression may occur via transcriptional interference or through competition for binding to shared enhancer elements. Moreover, Rom et al. have revealed that CHD2 binds the CHASERR nascent transcript and promotes gene expression through this interaction.

Heterozygous loss of CHASERR in mice results in increased neonatal mortality between days 1 and 4, leading to growth retardation, shorter lifespans, and impaired morphology in various organs. Homozygous deletions of CHASERR have so far only been obtained in cell cultures, while mouse models are not viable (5). Recently, three cases of heterozygous *de novo* deletion at the CHASERR locus caused by Alu-mediated nonallelic homologous recombination have been reported (6). Heterozygous loss of CHASERR in humans leads to developmental delay, facial dysmorphism, and cerebral hypomyelination.

Several studies have identified potential mechanisms of CHASERR function, in addition to its role in CHD2 repression. Liu et al. (7) demonstrated that CHASERR promotes colon cancer metastasis by recruiting EZH2 to the NFKBIB promoter, forming a positive feedback loop. Antonov et al. (8) proposed a *trans*-regulatory model wherein CHASERR interacts with nascent transcripts and directs the CHD2 helicase to target gene promoters. Wu et al. (9) demonstrated that m6A-modified lncRNA CHASERR promotes glioma growth and metastasis by sponging miR-6893-3p to upregulate TRIM14 expression.

Rom et al. (5) have noted that according to ENCODE and FANTOM5 datasets, CHD2 and CHASERR expression is particularly high in lymphocytes. The cell-type-specific expression pattern of CHASERR in lymphocytes strongly implies its participation in immune mechanisms. The key objectives of this study were to pinpoint the immune cell types subject to CHASERR-mediated regulation and to predict its involved pathways, an aim we pursued by analyzing its expression landscape using single-cell RNA-seq of PBMCs and its temporal dynamics using bulk RNA-seq during early T cell activation.

## 2 Results

### 2.1 LncRNA CHASERR is upregulated in naïve, central memory, and regulatory T cells compared to other immune cell types

**Figure 1:**
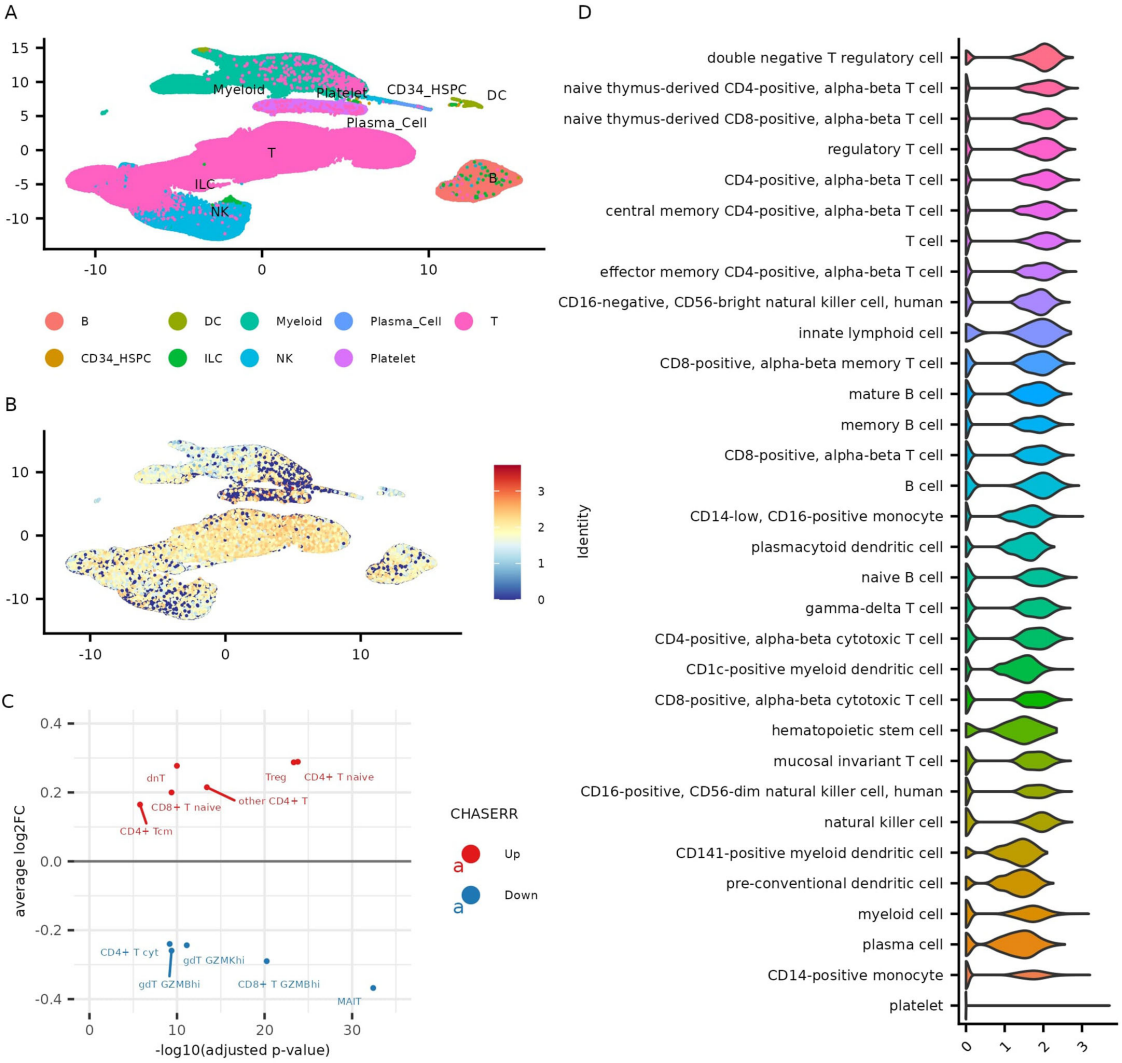
CHASERR expression across cell types in the AIDA dataset. **(A)** Gene expression UMAP of the AIDA dataset. **(B)** Normalized expression of CHASERR. **(C)** Wilcoxon test results for T cell subtypes of the AIDA dataset in pseudobulk profiles grouped by donors (one against all comparison, adjusted p-value *<* 0.05). **(D)** CHASERR expression in different cell types sorted in descending order of mean expression.

To define the expression landscape of the CHASERR–CHD2 axis across human immune cells, we analyzed single-cell RNA sequencing (scRNA-seq) data from the Asian Immune Diversity Atlas (AIDA), a comprehensive dataset of 1,265,624 peripheral blood mononuclear cells (PBMCs) (10). We found that CHASERR expression was significantly higher in T cells than in B or NK cells (adjusted *p <* 0.05; **Supplementary File 2**). Among T cell subsets, the highest expression levels were detected in double-negative regulatory T cells, naïve T cells, and regulatory T cells (Treg), while the lowest expression was observed in gamma-delta T cells and mucosal-associated invariant T (MAIT) cells (**Figures 1A–C**). Further stratification revealed that CHASERR expression was higher in central memory (TCM) compared to effector memory (TEM) T cells in both CD4+ and CD8+ lineages (**Figure 1D**). Among monocytes, CHASERR was more highly expressed in CD16+ than in CD14+ subsets.

As expected from its known repressive role, CHD2 expression often exhibited an inverse relationship with CHASERR—most notably in Tregs and double-negative T cells, where CHD2 levels were significantly lower (**Supplementary File 1: Supplementary Figures S1A–D**). However, in many other cell types, including naïve CD4+ and CD8+ T cells, CHD2 expression mirrored that of CHASERR, suggesting both repressive and co-regulated modes of interaction depending on cellular context. Indeed, although CHASERR and CHD2 were positively correlated across most cell types, their divergent behavior in Tregs and monocytes implies complex, cell-type-specific regulation.

**Figure 2:**
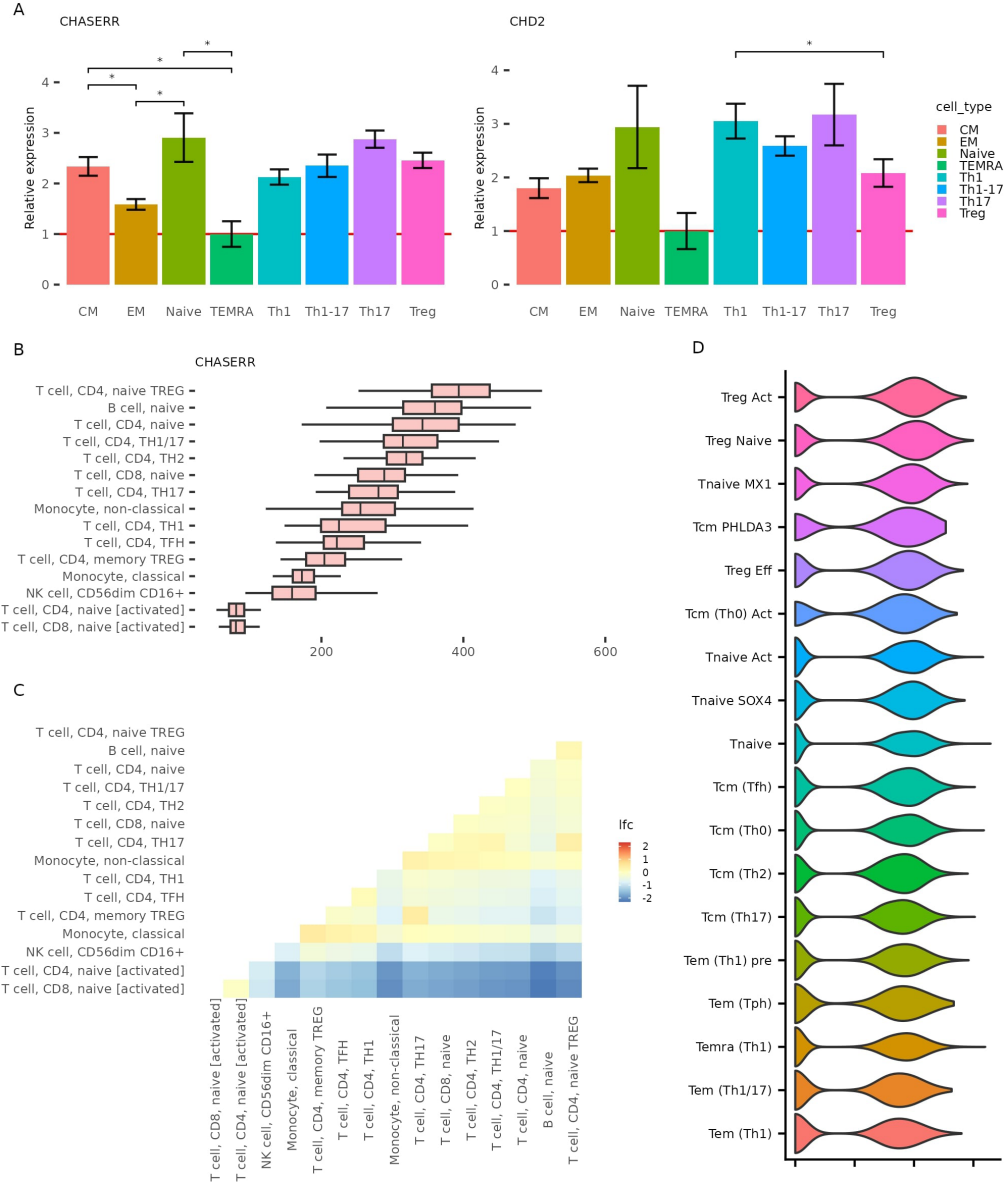
CHASERR and CHD2 expression across validation datasets. **(A)** CHASERR and CHD2 expression obtained in qPCR in CD4+ T cell subtypes isolated by FACS and the results of the T test pairwise comparison in two groups: Naive, CM, EM, TEMRA and Th1, Th17, Th1-17, Treg (*p *<* = 0.05, **p *<* = 0.001, ***p *<* = 0.0001, ****: p *<* = 0.00001). **(B)** CHASERR expression in immune cell types in DICE database (TPM) (11). **(C)** Differential expression analysis results for pairwise comparison between cell types for CHASERR in DICE dataset (FDR *<* 0.05). **(D)** CHASERR expression in CD4+ T dataset (12) with the second level annotation, sorted in descending order of mean expression.

We next sought to validate these findings experimentally. Using fluorescence-activated cell sorting (FACS), we isolated populations of primary human CD4+ T cell subsets—naïve, TCM, TEM, TEMRA, Th1, Th1-17, Th17, and Tregs—and quantified CHASERR and CHD2 expression via qPCR (**Figure 2A**). Consistent with the AIDA data, both genes were most highly expressed in naïve T cells. We also confirmed divergent expression patterns: CHASERR was elevated in TCM relative to TEM cells, while CHD2 showed the opposite trend. Among helper T subsets, CHASERR was highest in Th17 and Tregs, whereas CHD2 was highly expressed in Th1 and Th17 but reduced in Tregs.

To ensure robustness, we turned to two independent public datasets. Analysis of the DICE database (11), which contains bulk RNA-seq from 13 immune cell types, confirmed that CHASERR is most highly expressed in naïve CD4+ T cells and naïve Tregs, with the lowest levels in activated T cells (**Figures 2B, C**). Notably, CHD2 showed less heterogeneity across T cell subsets. We also analyzed scRNA-seq data from CD4+ T cells in healthy and autoimmune donors (12), which again revealed elevated CHASERR expression in naïve and Treg populations, with the highest levels in activated Tregs (**Figure 2D**, **Supplementary File 1: Supplementary Figures S2C, D**).

Together, these results firmly establish that CHASERR is most abundant in naïve, regulatory, and central memory T cells, and reveal both concordant and antagonistic expression patterns with its target, CHD2, across immune cell subtypes.

### 2.2 CHASERR is most highly expressed in Treg and Th17 cells among CD4+ T cell subsets

**Figure 3:**
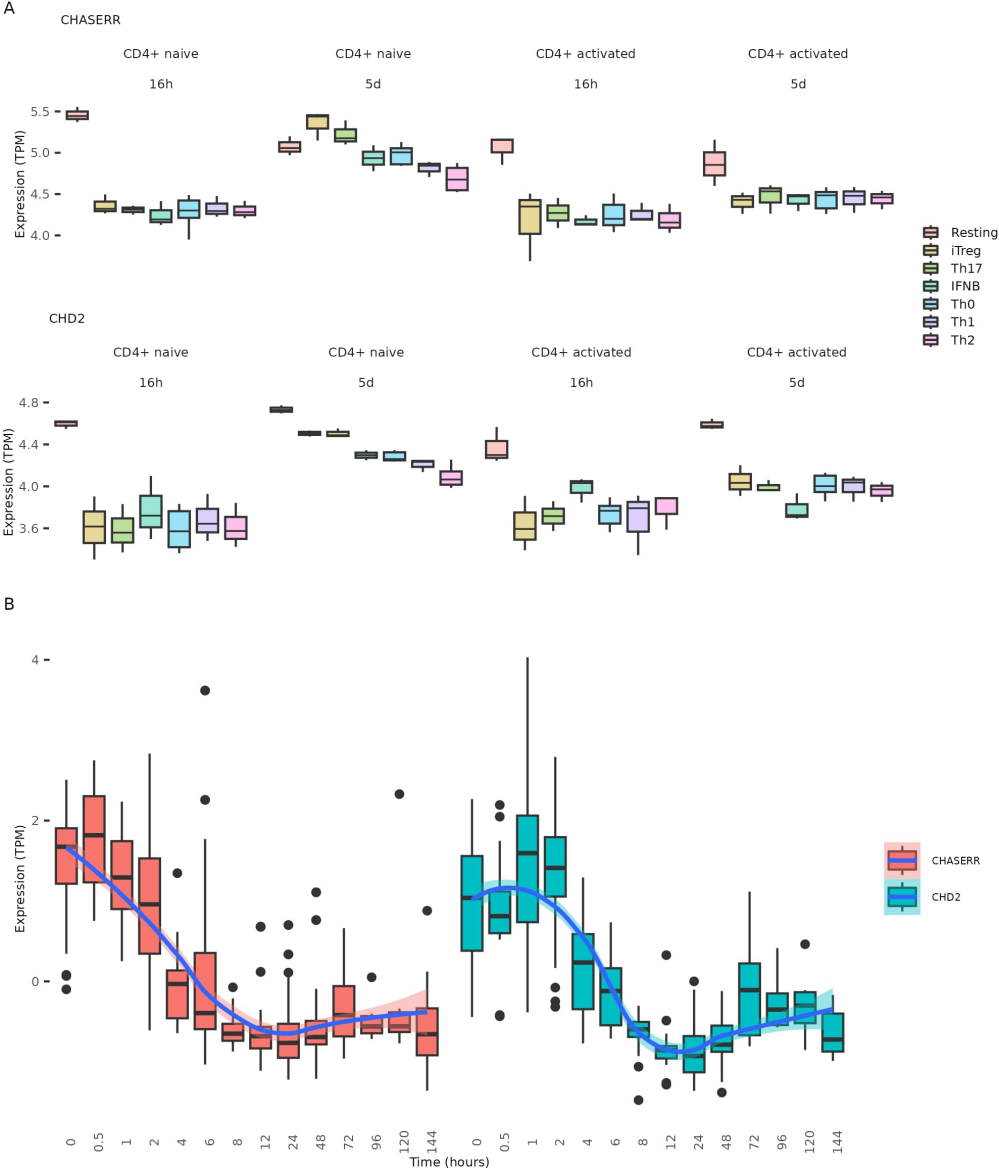
CHASERR and CHD2 expression changes during CD4+ T polarization and T cell activation. **(A)** CHASERR and CHD2 expression (TPM) from the CD4+ T polarization dataset (13). **(B)** CHASERR and CHD2 expression (TPM) at time points after T cell activation for cells from the T cell activation datasets (15).

To further investigate CHASERR expression patterns across CD4+ T cell subtypes, we analyzed bulk RNA-seq data from naïve and memory CD4+ T cells subjected to five distinct polarization conditions using different cytokine combinations (13). After 16 hours of polarization, CHASERR expression decreased in both naïve and memory T cells across all cytokine conditions. However, following five days of polarization, naïve T cells showed increased CHASERR expression in Treg and TH17 cells compared to both resting cells and cytokine-free controls (**Figure 3A**). CHD2 expression exhibited a similar pattern in polarized naïve T cells at day 5, though in iTregs and Th17 cells, CHD2 levels did not exceed those observed in resting cells. The marked variability in CHASERR and CHD2 expression across polarized naïve CD4+ T cell subtypes, coupled with consistently low expression in memory cells, suggests that their transcription may be repressed in memory T cells while remaining plastic in naïve populations.

We next examined whether CHASERR and CHD2 might be regulated by FOXP3, a master transcriptional regulator of Treg cell differentiation (14). In human Treg cells, FOXP3 ChIP-seq analysis revealed a binding peak within 2,000 base pairs of the CHASERR transcription start site (TSS) (**Supplementary File 1: Supplementary Figure S3A**). In mouse Treg cells, Foxp3 bound directly to both the Chd2 and Chaserr promoter regions (**Supplementary File 1: Supplementary Figure S5B**). Additionally, Foxp1, another Foxp family member essential for T cell quiescence and differentiation, was found to occupy the promoter regions of both Chaserr and Chd2 in mouse Treg cells and spleen CD8+ T cells (**Supplementary File 1: Supplementary Figure S3B**).

### 2.3 CHD2 upregulation follows CHASERR decrease during early stages of T cell activation

The elevated expression of both CHASERR and CHD2 in naïve compared to effector T cells prompted us to investigate their temporal dynamics during T cell activation. We analyzed six bulk RNA-seq datasets from a meta-analysis by Rade et al. (15), in which T cells were activated via anti-CD3/anti-CD28 antibodies.

Notably, CHASERR expression began to decline immediately following T cell activation (**Figure 3B**, **Supplementary File 1: Supplementary Figure S4**). In contrast, CHD2 expression exhibited a transient increase, peaking approximately one hour post-activation before gradually declining (**Supplementary File 1: Supplementary Figure S5**). This sequential pattern—CHASERR downregulation followed by transient CHD2 upregulation—suggests that the CHASERR-CHD2 axis may serve as an early regulatory module in T cell activation.

To identify potential functional targets of this axis, we examined whether genes altered upon CHASERR knockdown in fibroblasts (FANTOM6 project (16)) were also differentially expressed during T cell activation (15). Several genes downregulated after CHASERR knockdown — including RICTOR, RBL2, and USP30 — were also suppressed during T cell activation. Conversely, genes upregulated upon knockdown, such as HN1 and OSBPL3, showed increased expression during activation (**Supplementary File 1: Supplementary Figure S6**). These overlapping signatures suggest that CHASERR may regulate a conserved set of targets across cell types, including T cells.

The specific functions of these genes further support their role in promoting T cell activation: upregulation of HN1, a regulator of cell proliferation and microtubule stability (17, 18), alongside downregulation of the cell cycle inhibitor RBL2 (19), likely facilitates exit from quiescence, proliferation, and cytoskeletal remodeling essential for T cell function.

### 2.4 CHASERR downregulation is linked to effector T cell function and loss of quiescence

**Figure 4:**
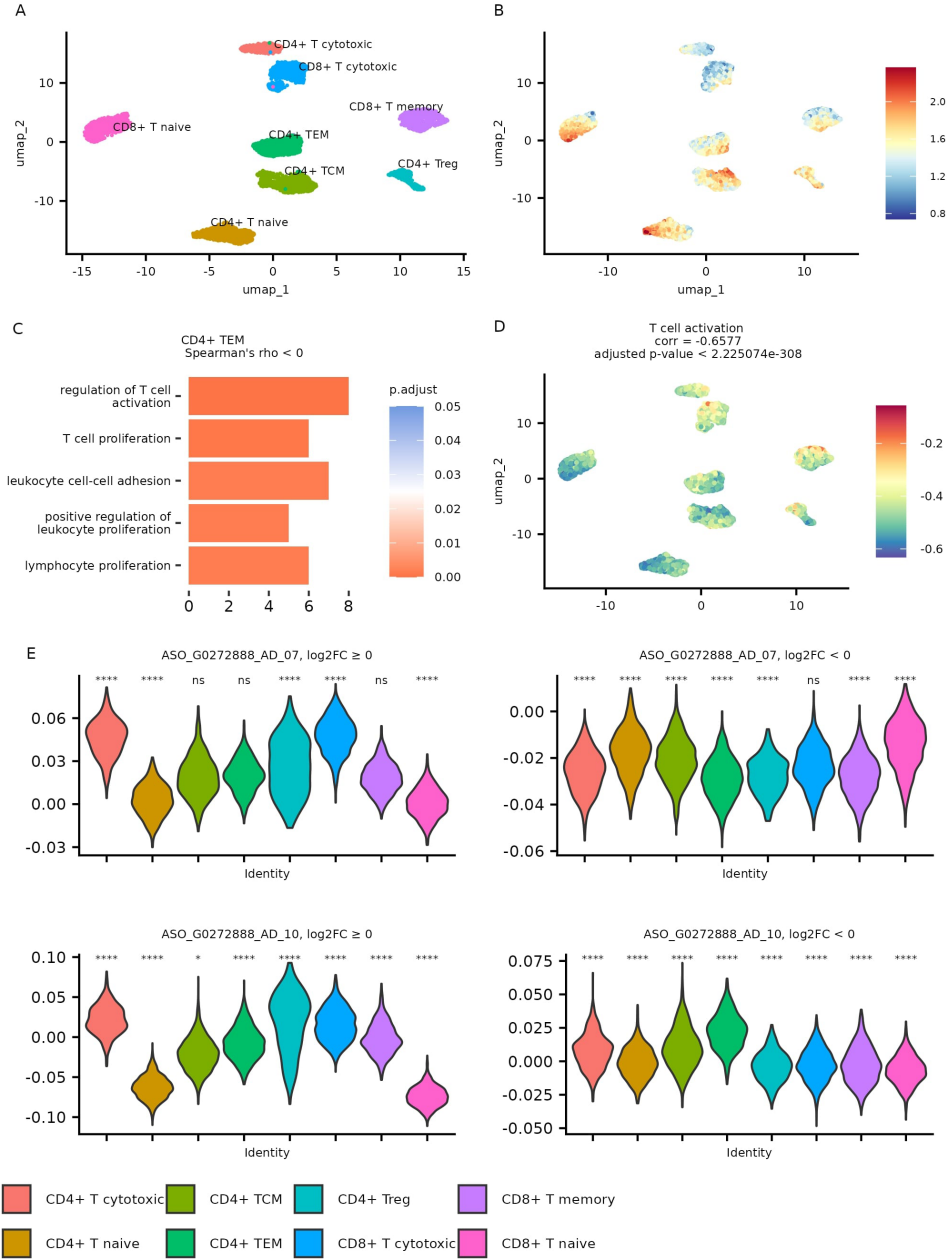
Metacell analysis of AIDA dataset T cells. **(A)** Gene expression UMAP for metacells. **(B)** CHASERR expression in metacells. **(C)** GO: BP pathways enriched in a set of genes with negative correlation with CHASERR in CD4+ TEM metacells (qvalue *<* 0.05). **(D)** T cell activation pathway (GO:0042110) scores in metacells. **(E)** FANTOM6 CHASERR knockdown signature scores for up- and down-regulated genes in T cell types across metacells and the results of Wicoxon test, each cell type is compared to all (ns: p *>* 0.01, *p *<* = 0.01, **p *<* = 0.001, ***p *<* = 0.0001, ****p *<* = 0.00001).

To explore potential functional relationships involving CHASERR in T cells, we performed co-expression analysis using metacells—aggregated cell states—generated from the AIDA dataset to address single-cell data sparsity. The CHASERR expression profile across metacells appeared generally consistent with patterns observed at the single-cell level (**Figures 4A, B**, **Supplementary File 1: Supplementary Figure S7**).

We identified genes showing correlation with CHASERR (Spearman’s |*ρ*| *>* 0.5, adjusted *p <* 0.05) across T cell subtypes (**Supplementary File 3**). Genes positively correlated with CHASERR included several regulators associated with quiescence, such as *BTG1*, *BTG2* (20), *TOB1* (21), *FOXP1* (22), and *ZFP36L2* (23). Genes negatively correlated with CHASERR in memory and effector T cells showed some enrichment in pathways related to effector functions, including proliferation, migration, and cytotoxicity (**Figure 4C**, **Supplementary File 4**). Some of these pathways included *CORO1A* (coronin-1A), an actin-binding protein that has been reported to facilitate Ca^2+^ mobilization (24) and may contribute to T cell survival (25).

We also computed Gene Ontology (GO) Biological Process activity scores for each metacell (**Supplementary File 5**). The T cell activation pathway showed a negative correlation with CHASERR expression (**Figure 4D**), which appears consistent with the pattern of higher CHASERR levels in quiescent naïve T cells and their reduction upon activation.

Along with the observed downregulation of CHASERR following T cell activation and the presence of cell cycle regulators among CHASERR-sensitive genes, these results may suggest a potential role for CHASERR in maintaining T cell quiescence. Its downregulation appears to coincide with the acquisition of effector functions, though further investigation would be needed to establish causal relationships.

### 2.5 CHASERR knockdown is associated with upregulation of genes active in cytotoxic and effector T cells

To explore potential functional consequences of CHASERR loss across T cell subtypes, we examined enrichment patterns for genes differentially expressed in FANTOM6 CHASERR knockdown experiments (FDR ¡ 0.05) within metacells from the AIDA dataset (**Figure 4E**).

Genes upregulated following CHASERR knockdown tended to show higher enrichment scores in cytotoxic T cell populations compared to naïve T cells. Meanwhile, genes downregulated after CHASERR knockdown (particularly in the ASO G0272888 AD 07 experiment) appeared most enriched in CD8+ naïve T cells. This pattern suggests a potential inverse relationship between CHASERR expression and the enrichment scores of genes responsive to its knockdown.

These observations raise the possibility that genes affected by CHASERR knockdown might represent functional targets in T cells, and that loss of CHASERR could potentially contribute to transcriptional states associated with cytotoxic and effector T cell function. However, further validation in T cell models would be needed to confirm this relationship.

### 2.6 Cyclosporine A mitigates transcriptional consequences of CHASERR deficiency

To identify potential therapeutic compounds that could counteract the effects of CHASERR loss, we screened the LINCS database using the transcriptional signature from FANTOM6 CHASERR knockdown experiments (ASO G0272888 AD 07; **Supplementary File 6**). Among the top-ranked candidates, we selected cyclosporine A (CsA) — a known immunosuppressant that forms a complex with cyclophilin to inhibit calcineurin, thereby preventing nuclear translocation of NFAT and T cell activation (26) - for experimental validation.

**Figure 5:**
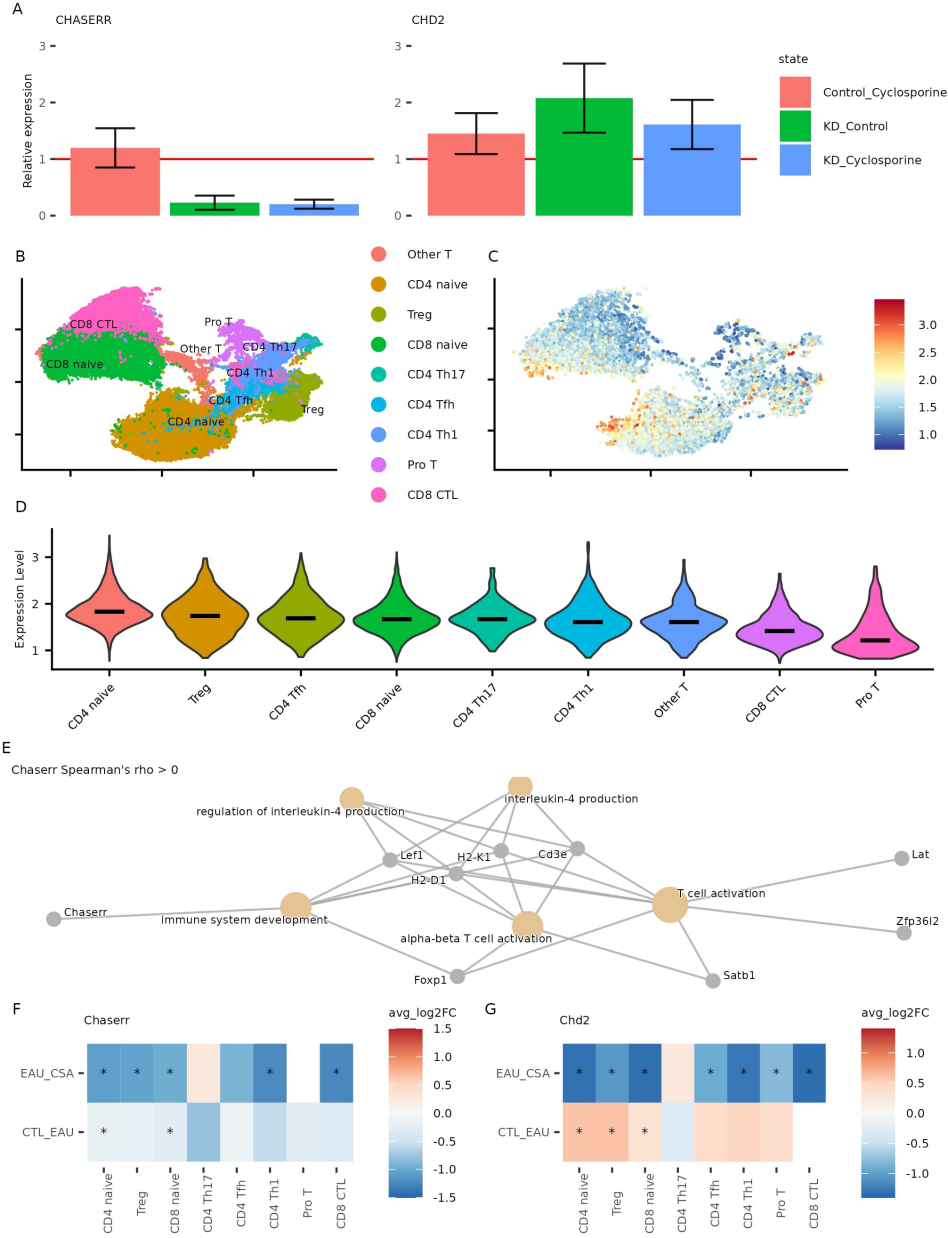
Effect of cyclosporine on CHASERR and CHD2 expression. **(A)** CHASERR and CHD2 expression obtained in qPCR for three replicates (replicate 1,3: after 24 hours, replicate 2: after 20 hours): untreated control (y = 1), CHASERR knockdown without treatment (KD Control), control with treatment (Control Cyclosporine), and knockdown with treatment (KD Cyclosporine). **(B)** Gene expression UMAP for T cells in the cyclosporine dataset (27). **(C)** Normalized expression of Chaserr in the cyclosporine dataset, (Chaserr expression *>* 0). **(D)** Chaserr expression in T cell subtypes. **(E)** GO: BP pathways enriched in a set of genes with positive correlation with Chaserr (qvalue *<* 0.05). **(F, G)** Wilcoxon test log fold change for Chaserr **(F)** and Chd2 **(G)** in each cell type comparing uveitis group (EAU) against control group (CTL) and comparing cyclosporine treatment group (CSA) against uveitis group (*adjusted p-value *<* 0.05).

We first tested whether CsA could modulate CHD2 expression in the context of CHASERR deficiency. Following CHASERR knockdown in primary human fibroblasts, we observed the expected increase in CHD2 expression by qPCR (**Figure 5A**), consistent with previous reports of CHASERR-mediated repression (5). While CsA treatment did not restore CHASERR expression itself — likely due to persistent ASO-mediated knockdown — it significantly reduced CHD2 expression in CHASERR-deficient cells. This provides only a preliminary indication that CsA may partially compensate for CHASERR loss by normalizing CHD2 levels through a mechanism independent of CHASERR re-expression in human.

To validate these findings in mouse model, we analyzed scRNA-seq data from lymph nodes of mice with experimental autoimmune uveitis (EAU) treated with CsA (27). Consistent with human data, Chaserr expression was highest in CD4+ naïve and regulatory T cells and lowest in CD8+ cytotoxic T cells (**Figures 5B–D**). We also confirmed conserved positive correlations between Chaserr and key quiescence factors Foxp1 and Zfp36l2 (adjusted *p <* 0.05; **Supplementary File 7**), and Gene Ontology analysis revealed enrichment of Chaserr-correlated genes in T cell activation pathways, including Satb1—a regulator of chromatin remodeling in T cells (28) (**Figure 5E**, **Supplementary File 8**).

In the EAU model, Chaserr expression was reduced across T cell subsets, and CsA treatment further decreased its expression (**Figure 5F**). In contrast, Chd2 levels were elevated in uveitis but reduced following CsA treatment (**Figure 5G**). These results align with our *in vitro* data and suggest that CsA can counteract Chd2 overexpression in settings of CHASERR deficiency, even in an active autoimmune context.

While CsA does not restore CHASERR expression, our findings indicate that it may mitigate key transcriptional consequences of CHASERR loss, particularly dysregulation of CHD2. This supports further investigation of calcineurin-NFAT pathway inhibitors as potential therapeutic strategies for conditions linked to CHASERR deficiency.

## 3 Methods

### 3.1 Data sources

To investigate the expression dynamics of CHASERR and CHD2 across immune cell populations, we analyzed 10 datasets:

AIDA dataset. AIDA Data Freeze v2 gene-cell matrix contains scRNA-seq data for adult PBMC (https://cellxgene.cziscience.com/collections/ced320a1-29f3-47c1-a735-513c7084d508) (10).
DICE dataset. The DICE database contains bulk RNA-seq data from 13 immune cell types (https://dice-database.org/) (11).
CD4+ T dataset. Droplet-based scRNA-seq data for PBMC cells from healthy donors and donors with autoimmune diseases (https://singlecell.broadinstitute.org/singlecell/study/SCP1963) (12).
CD4+ T polarization dataset. Bulk RNA sequencing data obtained during the polarization of memory and naïve CD4+ T cells (https://www.opentargets.org/projects/effectorness) (13).
Cyclosporine dataset. Cyclosporine treatment scRNA-seq FASTQ files were downloaded from the Genome Sequence Archive accession CRA006097 (27).
T cell activation datasets. Six bulk RNA-seq datasets contain expression counts for T cells in the early stages of activation: GSE90569 (29), GSE96538 (30)), GSE94396 (30), GSE52260 (31), GSE140244 (32), and GSE197067 (15).
FANTOM6 dataset. Differentially expressed genes (FDR *<* 0.05) identified in CHASERR knockdown experiments (ASO_G0272888_AD_07 and ASO_G0272888_AD_10) were downloaded from the FANTOM6 project (https://fantom.gsc.riken.jp/6/datafiles/Core_FANTOM6/RELEASE_latest/analysis/DEGs/01_combined/DE (16)).
FOXP3 ChIP-seq human. ChIP-seq FASTQ data for FOXP3 in human Treg cells were downloaded from GSE43119 (33).
Foxp1 and Foxp3 ChIP-seq Treg mouse. ChIP-seq peaks corresponding to Foxp1 and Foxp3 in mouse Treg cells (34).
Foxp1 ChIP-seq CD8+ mouse. ChIP-seq FASTQ data for Foxp1 in mouse spleen CD8+ T cells were downloaded from GSE202543 (35).

### 3.2 Flow cytometry and cell sorting

To identify and sort naive and memory CD4+ T cell subsets, PBMCs (510^6^ cells in 100) were stained with a cocktail of fluorescently labeled antibodies: CD3-BB700 (clone OKT3, 1:20 dilution), CD4-BV786 (clone OKT4, 1:20), CD45RA-BV480 (clone HI100, 1:20), and CD197 (CCR7, clone G043H7, 1:20). Naïve CD4+ T cells were defined as CD45RACCR7, central memory (CM) as CD45RA-CCR7-, effector memory (EM) as CD45RA-CCR7-, and terminally differentiated effector memory (TEMRA) as CD45RA+CCR7-. A representative gating strategy is provided in **Supplementary File 1: Supplementary Figures S8A1–A6**.

For the sorting of Treg and Th1/Th17 subsets, PBMCs (510^6^ cells in 100) were stained with a separate antibody panel: CD3-BB700 (clone OKT3, 1:20), CD4-BV786 (clone OKT4, 1:20), CD183-BV421 (CXCR3, clone G025H7, 1:20), CD196-BB515 (CCR6, clone 11A9, 1:20), CD127-AF647 (clone HIL-7R M21, 1:20), and CD25-PE (clone M-A251, 1:20). The gating strategy is shown in **Supplementary File 1: Supplementary Figures S9A1–A5**.

All antibodies were obtained from BD Biosciences (USA), except CD196 and CD183, which were sourced from BioLegend (USA). Cell sorting was performed on a BD FACS Aria III sorter equipped with 405, 488, 561, and 633 nm lasers. Compensation was carried out using anti-mouse Ig compensation beads (BD Biosciences) stained with respective antibodies, and compensation matrices were automatically calculated using BD FACSDiva software (v9.0.1). Cells were sorted in purity mode into 5 mL tubes. Postsort validation was performed by reanalyzing 100 µL of sorted cells mixed with 100 PBS (**Supplementary File 1: Supplementary Figures S8B1–B4, S9B1–B4**). Final sorted cell counts were as follows: 336,500 naive cells, 276,000 CM cells, 170,500 EM cells, 33,590 TEMRA cells, approximately 50,000 Tregs, 177,000 Th1 cells, 57,500 Th17 cells, and 101,780 Th1–17 cells.

### 3.3 Quantitative real-time PCR analysis of FACS-sorted CD4+ T cell subsets

RNA was extracted by direct cell lysis with ExtractRNA reagent (Evrogen). 5 ng of total RNA was Total RNA was extracted from sorted CD4+ T cell populations by direct lysis using ExtractRNA reagent (Evrogen). For cDNA synthesis, 5 ng of total RNA was reverse transcribed using the MMLV RT kit with dT20 primer (Evrogen). Quantitative real-time PCR was performed using 5X SYBR Green master mix (Evrogen). Gene expression levels were normalized to the housekeeping gene *B2M* and analyzed using the ΔΔCt method (36). All primer sequences are provided in **Supplementary File 9**.

### 3.4 Single-cell RNA-seq data processing

All scRNA-seq data were processed using Seurat v4.3.3 (37). For the cyclosporine dataset, reads were aligned to the GRCm39 reference genome using CellRanger v8.0.1 (https://support.10xgenomics.com) with Ensembl v111 annotation. Quality control filtering excluded cells with <200 or >2500 detected genes, total UMI counts <500 or >10,000, or mitochondrial gene content >15%. Genes detected in <1% of cells were removed. Count normalization was performed using the LogNormalize function in Seurat. Following normalization and scaling, we performed nearest-neighbor analysis, Louvain clustering, and batch correction via Harmony integration (38). T cell clusters were identified by expression of *Cd3e*, *Cd3d*, and *Cd3g*, with further subclustering using established marker genes (**Supplementary File 1: Supplementary Figure S10**) (39).

### 3.5 Differential expression analysis in bulk and single-cell RNA-seq datasets

Differential expression analysis for the AIDA dataset was performed by comparing each T cell type against all other cell types from the layer 1 annotation using the FindMarkers function in Seurat. We applied the Wilcoxon rank-sum test with return.thresh = 1 and calculated adjusted p-values using Bonferroni correction.

For subtype-specific analyses within T cells in both the AIDA and CD4+ T datasets, we identified differentially expressed genes between each T cell subtype and all other T cell subtypes using the FindAllMarkers function in Seurat with Wilcoxon rank-sum tests (return.thresh = 1) and Bonferroni-adjusted p-values. Additionally, we performed differential expression analysis on pseudobulk expression profiles aggregated by donor and cell type using the same statistical approach.

Bulk RNA-seq differential expression results for the DICE database were obtained from the DICE Portal (https://dice-database.org/) (11). These analyses were performed using DESeq2 (40), and we retained only results with an adjusted p-value *<* 0.05 (Benjamini Hochberg method).

Differential expression analysis was conducted on T cell activation datasets (GSE90569, GSE96538, GSE94396, GSE52260, GSE140244). Gene expression at the 1-hour and 2-hour time points was compared against the 0-hour control. The analysis was performed using DESeq2 (40), and p-values were adjusted for multiple testing using the Benjamini-Hochberg method.

For the cyclosporine treatment dataset, we compared gene expression between the control and uveitis groups, as well as between the uveitis and cyclosporine-treated groups, within each annotated cell type using the FindMarkers function in Seurat (Wilcoxon rank-sum test, adjusted p-value *<* 0.05).

### 3.6 Metacell aggregation and coexpression analysis

Metacells were constructed from T cells in the AIDA dataset using hdWGCNA v0.4.0 (41). Libraries with mean CHASERR expression in the lowest 5% were excluded from analysis. Metacells were generated by mean aggregation of normalized counts using *k* = 75 nearest neighbors, with a maximum of 10 shared cells between any two metacells. Harmony integration was applied using the library_uuid covariate. The final analysis excluded generalized ‘CD4-positive, alpha-beta T cell’ and ‘CD8-positive, alpha-beta T cell’ annotations to focus on specific T cell subtypes.

Pairwise gene expression correlations were computed using Spearman’s rank correlation on aggregated, normalized expression vectors. Correlations with |*ρ*| *>* 0.5 and Bonferroni-adjusted p-values *<* 0.05 were retained for further analysis. Genes showing significant correlations underwent Gene Ontology enrichment analysis (Biological Process category) using clusterProfiler v4.14.3 (42), with all detected genes serving as the background set and a significance threshold of q-value *<* 0.05.

Module scores for genes upregulated and downregulated in FANTOM6 CHASERR knockdown experiments were calculated using the AddModuleScore function in Seurat. Gene Set Variation Analysis (GSVA) v2.0.5 (43) was used to compute GO Biological Process pathway scores for each metacell. Spearman correlations between GSVA pathway scores and CHASERR expression levels were calculated, retaining only results with Bonferroni-adjusted p-values *<* 0.05.

### 3.7 ChIP-seq data processing

We processed Foxp1 ChIP-seq data from mouse spleen CD8+ T cells from raw FASTQ files using the nf-core/chipseq pipeline (44). Reads were aligned to the mm10 reference genome using BWA v0.7.17-r1188 (45). Peak calling was performed with MACS3 v3.0.3 (46) (q-value *<* 0.05) for each biological replicate, and replicates were merged using the Irreproducible Discovery Rate (IDR) framework v2.0.4.2 (47). FOXP3 ChIP-seq data from human Treg cells (GSE43119) were processed using the same nf-core/chipseq pipeline parameters, with alignment to the hg38 genome. ChIP-seq peaks were visualized using the IGV browser (https://igv.org/app/).

### 3.8 Prediction of drugs reversing CHASERR knockdown effects

To identify compounds capable of counteracting the transcriptional signature of CHASERR knockdown, we submitted differentially expressed genes (FDR *<* 0.05) from the FANTOM6 CHASERR knockdown experiment (ASO_G0272888_AD_07) to the L1000FWD platform (https://maayanlab.cloud/l1000fwd/) (48). Candidate drugs were prioritized based on combined scores, with emphasis on compounds whose gene expression signatures were inversely correlated with the CHASERR knockdown phenotype.

### 3.9 Quantitative real-time PCR validation of cyclosporine effects following CHASERR knockdown

Human dermal fibroblasts (HDFb d75, adult female) were obtained from the Cell Culture Collection (IDB RAS, Russia). Cells between passages 5–10 were maintained at 37 °C with 5% CO 2 in DMEM/F12 medium (Gibco) supplemented with 1×Penicillin-Streptomycin (Paneco) and 10% v/v FBS (BioWest). For lncRNA knockdown, cells at 75–80% confluency were transfected with ASOs targeting CHASERR using FectoMEM transfection medium (Bioinnlabs) and GenJect40 transfection reagent (Molecta) at a final concentration of 10 pmol ASO per cm^2^ growth surface. Total RNA was isolated by direct cell lysis with ExtractRNA reagent (Evrogen). Reverse transcription was performed on 50 ng of total RNA using MMLV RT kit with dT 20 primer (Evrogen). Quantitative PCR was carried out using 5× SYBR Green master mix (Evrogen). Three biological replicates were analyzed to assess cyclosporine effects—expression was measured at 20 hours post-treatment in the first experiment and at 24 hours in subsequent replicates. All experiments were analyzed independently using the ΔΔCt method (36) with normalization to *PPIA*. ASO sequences and qPCR primers are provided in **Supplementary File 9**.

## 4 Discussion

Long non-coding RNAs exhibit highly tissue-specific expression patterns (49), with many functioning in restricted cellular contexts, including specific immune cell subtypes (50). Our study focuses on CHASERR, a lncRNA with pronounced lymphocyte-specific expression (5) whose functional role in immunity remained poorly characterized. We demonstrate that CHASERR shows preferential expression in T cells compared to other immune populations, with particularly high levels in naïve T cells, Treg, and double-negative regulatory T cells. The inverse correlation between CHASERR knockdown signatures and its baseline expression patterns across T cell subsets suggests that loss of CHASERR may disrupt normal T cell functionality.

Regulatory T cells are critical components of the adaptive immune system and play a central role in preserving immunological self-tolerance. Impairment of Treg-mediated regulation leads to autoimmune disorders (51). Loss of CHASERR could potentially shift T cells toward effector phenotypes and cause Treg loss of function, thereby promoting autoimmune pathogenesis. Therefore, defining the impact of the CHASERR-CHD2 regulatory axis on the T cell effector functions and on differentiation into T helper types, including Treg cells, represents an important future endeavor.

Our ChIP-seq analyses reveal that both FOXP3 and Foxp1 bind to regulatory regions of CHASERR and CHD2 in human and mouse T cells. The strong positive correlation between FOXP1 and CHASERR expression across T cell subsets is particularly interesting given an established role of FOXP1 in maintaining T cell quiescence by suppressing IL-7R*α* expression and inhibiting antigen-independent proliferation (22). The graded expression of FOXP1 across T cell differentiation stages—highest in naive cells, reduced in central memory, and minimal in effector memory populations (52)—closely mirrors the expression pattern of CHASERR. Furthermore, the ability of FOXP1 to reinforce FOXP3 dependent transcriptional regulation (34) suggests potential cooperative interactions between FOXP1, FOXP3, and CHASERR in establishing both T cell quiescence and Treg identity.

We observed dynamic CHASERR and CHD2 expression changes during T cell activation, with different expression decline patterns upon stimulation. To identify potential CHASERR targets, we intersected genes differentially expressed upon T cell activation with those altered following CHASERR knockdown. This identified cell cycle regulators like HN1 and RBL2. However, their expression dynamics during activation could be either a consequence of CHASERR activity or a general correlate of T cell proliferation, making a direct causal link difficult to establish.

To assess whether pharmacological agents can rescue the phenotypic consequences of CHASERR loss, we linked the CHASERR knockdown transcriptomic signature to drug candidates. Cyclosporine, a top predictor, reduced CHD2 expression in fibroblasts with CHASERR knockdown and in murine models of autoimmune uveitis. In both our CHASERR knockdown model and in cyclosporine-treated autoimmune uveitis mice, we observed a consistent decrease in CHD2 levels without a corresponding increase in CHASERR expression. This suggests that cyclosporine treatment reduces CHD2 expression through mechanisms independent of CHASERR upregulation. These findings imply that cyclosporine may counteract downstream effects of CHASERR loss, highlighting its potential for modulating CHD2-mediated pathways in autoimmune contexts. The observed decrease in CHD2 expression in response to cyclosporine may indicate that CHD2 is a target gene of the calcineurin pathway, potentially activated by the nuclear translocation of NFAT.

A key limitation of our study is that the CHASERR knockdown was performed in fibroblasts rather than in primary T cells. This decision was necessitated by the well-documented technical challenges associated with achieving efficient transfection in primary T cells (53). Consequently, while our correlation analyses are robust and supported by multiple datasets, we can only infer a functional relationship rather than establish a direct causal link within a T cell context. Furthermore, the effect of CHASERR knockdown was assessed in cells from only one donor. Expanding this analysis to a larger cohort of donors is necessary to evaluate the influence of inter-individual variability on cyclosporine effects. To definitively confirm the role of the CHASERR-CHD2 axis in T cell quiescence and activation, future studies employing CHASERR knockdown or knockout in primary T cells from several donors will be essential.

## 5 Conclusion

Our results implicate the long non-coding RNA CHASERR and its target gene CHD2 in the regulation of immune response. We found that both CHASERR and CHD2 are highly expressed in T cells — particularly in naive T cells — with further upregulation of CHASERR in regulatory T cells. Their elevated expression in naive cells, combined with dynamic changes during early T cell activation, suggests their involvement in maintaining T cell quiescence and anti-autoimmune phenotype.

Using data on gene expression in CHASERR knockdown, we identified cyclosporine — an immunosuppressant drug — as a potential therapeutic agent capable of counteracting CHASERR loss. Experimental validation by qPCR and the analysis of a cyclosporine treatment dataset confirmed its ability to suppress CHD2 expression.

While our work maps the expression dynamics of CHASERR and CHD2 across T cell subsets and provides comprehensive correlation analyses, definitive functional validation — such as cell-type-specific CHASERR knockdown in T cells — remains essential. Our findings establish a foundation for unraveling the CHASERR–CHD2 axis in immune homeostasis and propose cyclosporine as a candidate modulator of this pathway.

## Data Availability

The original contributions presented in the study are included in the article/**Supplementary Material**. Further inquiries can be directed to the corresponding author. The code used in data analysis is accessible in the GitHub repository https://github.com/lab-medvedeva/chaserr_immune and ZENODO https://doi.org/10.5281/zenodo.17238476 under MIT license.

## Supplementary material

Supplementary File 1 Supplementary Figures. **Figures S1** to **S10** with supporting information referenced in the main text.

Supplementary File 2 XLSX. Wilcoxon test results comparing CHASERR and CHD2 expression between T cells and other cell types.

Supplementary File 3 XLSX. Spearman rho values and adjusted p-values for correlation between CHASERR and other genes in the AIDA metacell dataset.

Supplementary File 4 XLSX. gene ontology biological process results for genes with significant correlation with CHASERR in the AIDA metacell dataset.

Supplementary File 5 XLSX. gene ontology biological process GSVA score correlation with CHASERR expression in the AIDA metacell dataset.

Supplementary File 6 XLSX. L1000fwd ranked list for ASO G0272888 AD 07 CHASERR knockdown gene signature.

Supplementary File 7 XLSX. Spearman rho values and adjusted p-values for correlation between Chaserr and other genes in T cells in the cyclosporine dataset.

Supplementary File 8 XLSX. gene ontology biological process results for genes with significant correlation with CHASERR in the cyclosporine dataset.

Supplementary File 9 XLSX. Primer and ASO sequences for qPCR.
