# Supplementary material for: CHASERR-CHD2 dynamics in T cell quiescence and its modulation by cyclosporine
Source: Front Immunol. 2025 Nov 11;16:1652359. doi: 10.3389/fimmu.2025.1652359 (PMC12644095; doi:10.3389/fimmu.2025.1652359)
Supplement: Supplementary File 1 — Supplementary Figures. Figures S1 to S10 with supporting information referenced in the main text. [file DataSheet1.pdf]

Supplementary Material

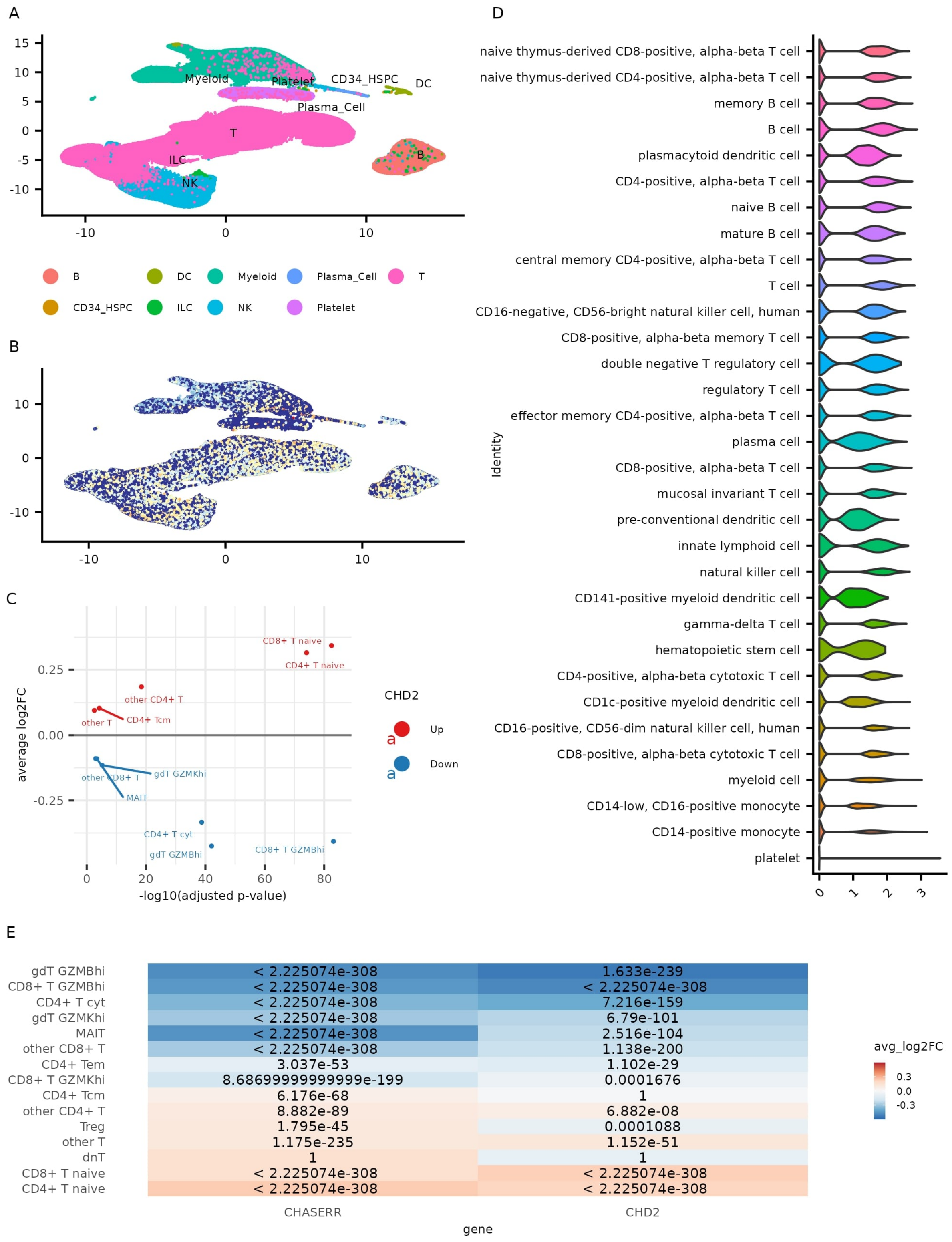

Figure S1. CHD2 expression across cell types in the AIDA dataset. **(A)** Gene expression UMAP of the AIDA dataset. **(B)** Normalized expression of CHD2. **(C)** Wilcoxon test results for T cell subtypes of the AIDA dataset in pseudobulk profiles grouped by donors (one against all comparison, adjusted p-value < 0.05). **(D)** CHD2 expression in different cell types sorted in descending order of mean expression. **E** Wilcoxon test results for T cell subtypes of the AIDA dataset (one against all comparison) without adjusted p-value threshold applied. The colour of the cell corresponds to the log2FC; the value in the cell corresponds to the adjusted p-value.

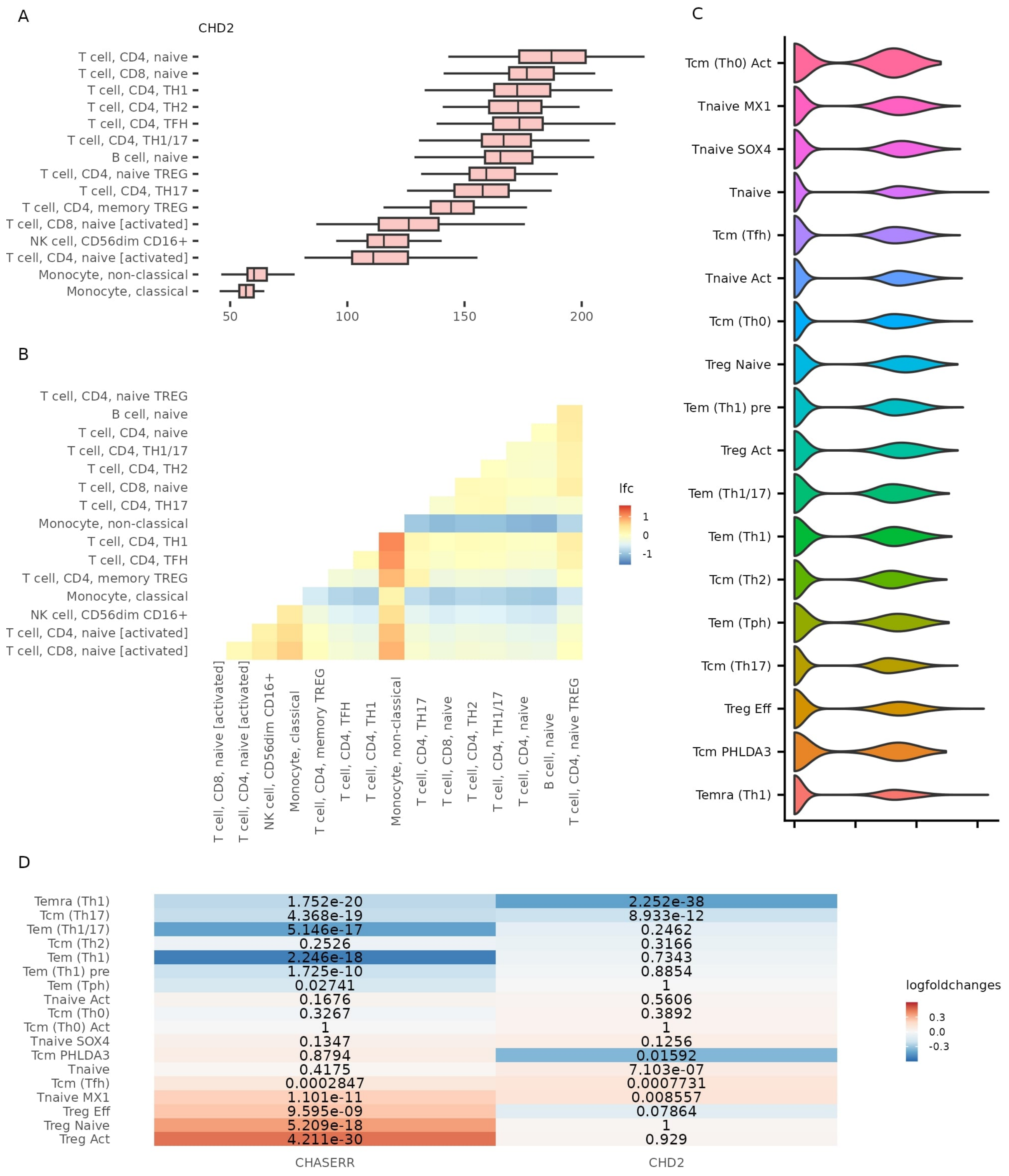

Figure S2. CHASERR and CHD2 expression across validation datasets. **A** CHD2 expression in immune cell types in DICE database (TPM). **B** Differential expression analysis results for pairwise comparison between cell-types for CHD2 in DICE dataset(FDR < 0.05). **C** CHD2 expression in CD4+ T dataset with the second level annotation, sorted in descending order of mean expression. **D** Wilcoxon test results for T cell subtypes of the CD4+ T dataset (one against all comparison) without adjusted p-value threshold applied. The colour of the cell corresponds to the log2FC; the value in the cell corresponds to the adjusted p-value.

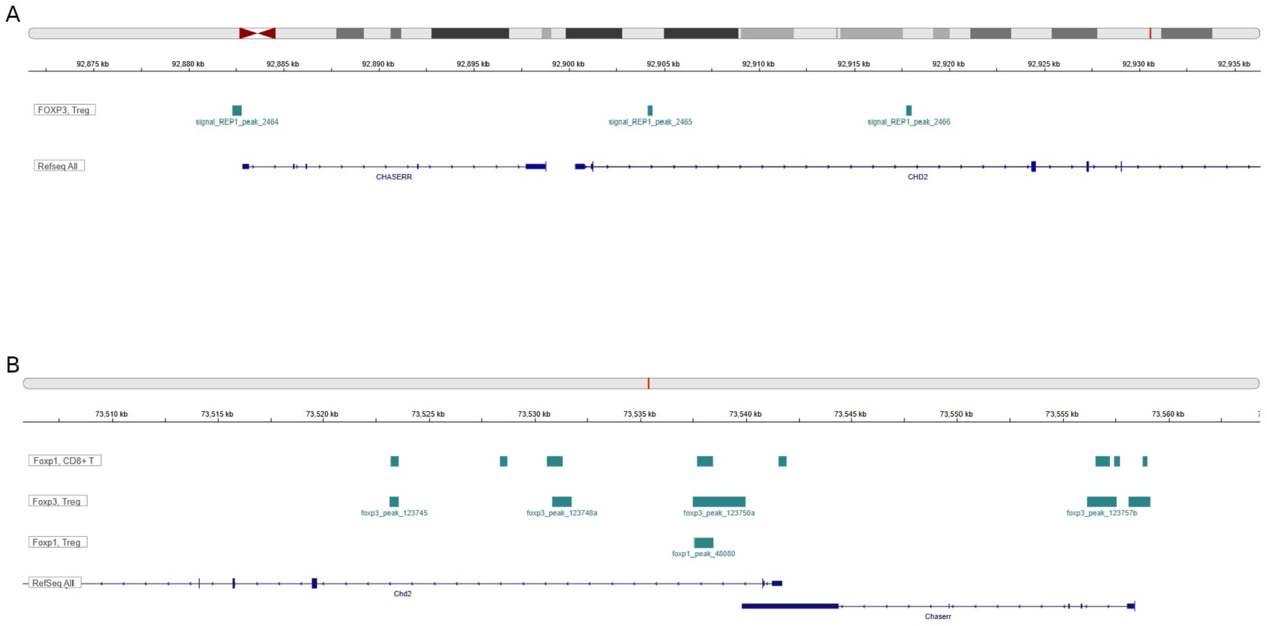

Figure S3. FOXP1 and FOXP3 binding sites from ChIP-seq experiments. **A** IGV browser visualization of FOXP3 ChIP-seq peaks in human Treg cells. **B** IGV browser visualization of Foxp1 and Foxp3 ChIP-seq peaks in mouse Treg and CD8+ T cells.

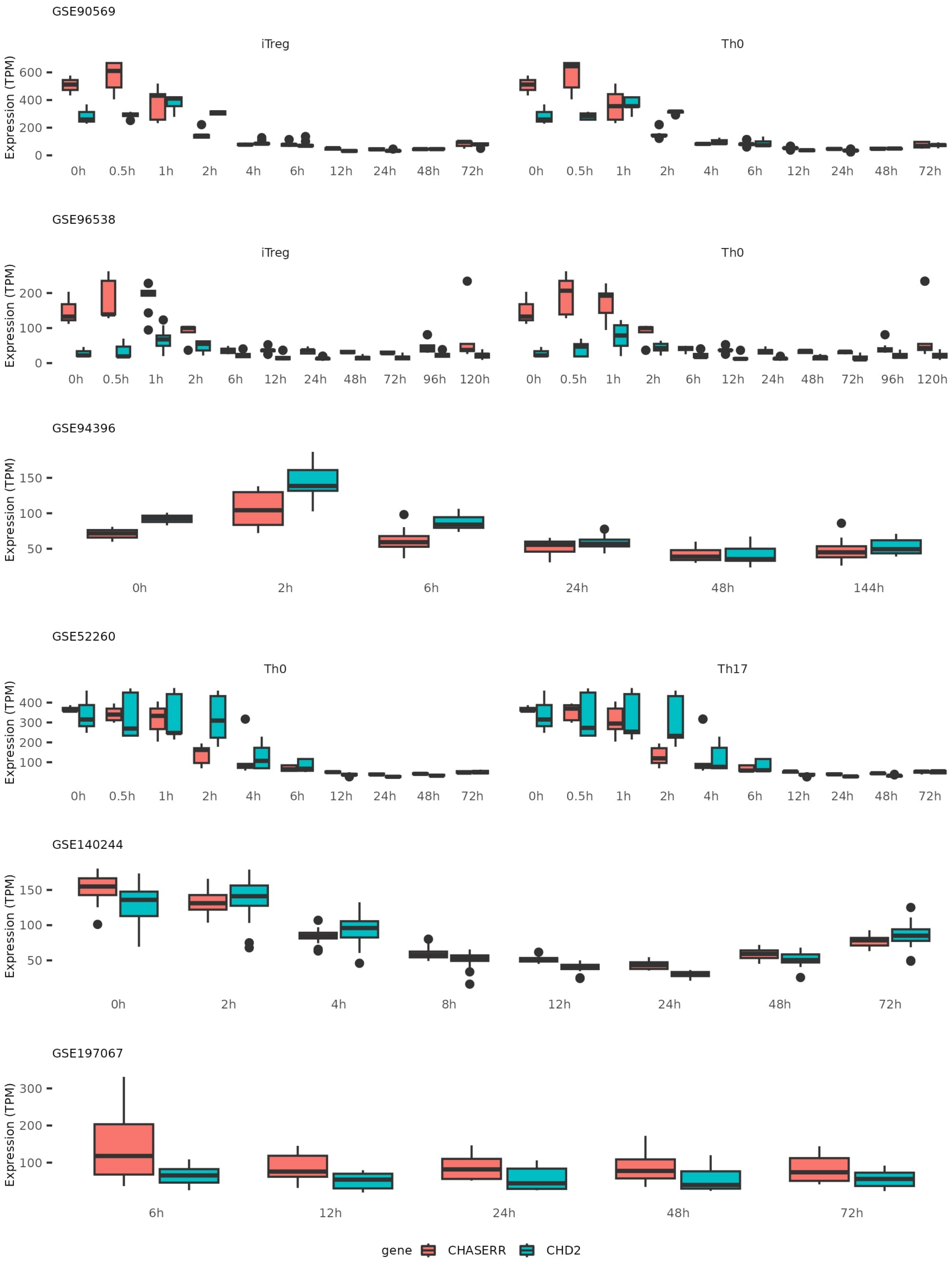

Figure S4. CHASERR and CHD2 expression (TPM) at time points after T cell activation from the T cell activation datasets.

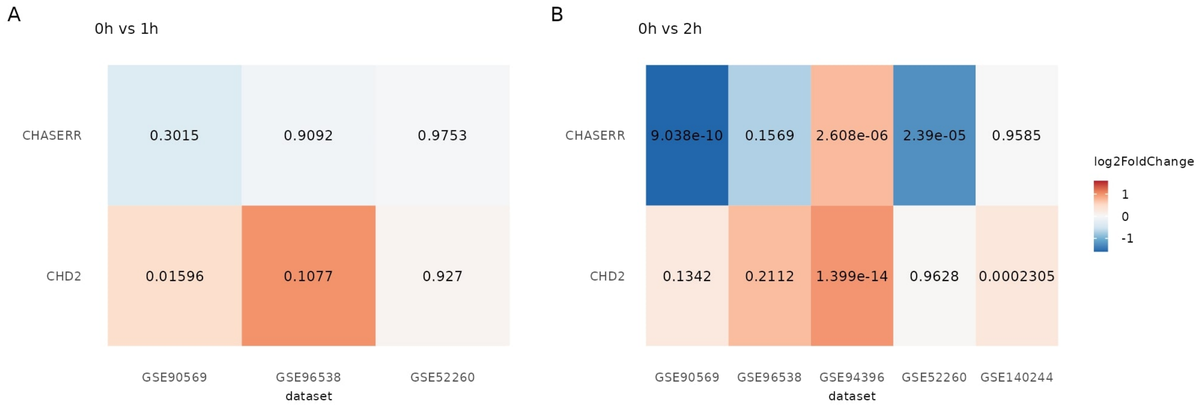

Figure S5. Differential expression analysis results for T cell activation time points 1h **(A)** and 2h **(B)** compared to 0h (DESeq2) without an adjusted p-value threshold applied. The colour of the cell corresponds to the log2FC; the value in the cell corresponds to the adjusted p-value.

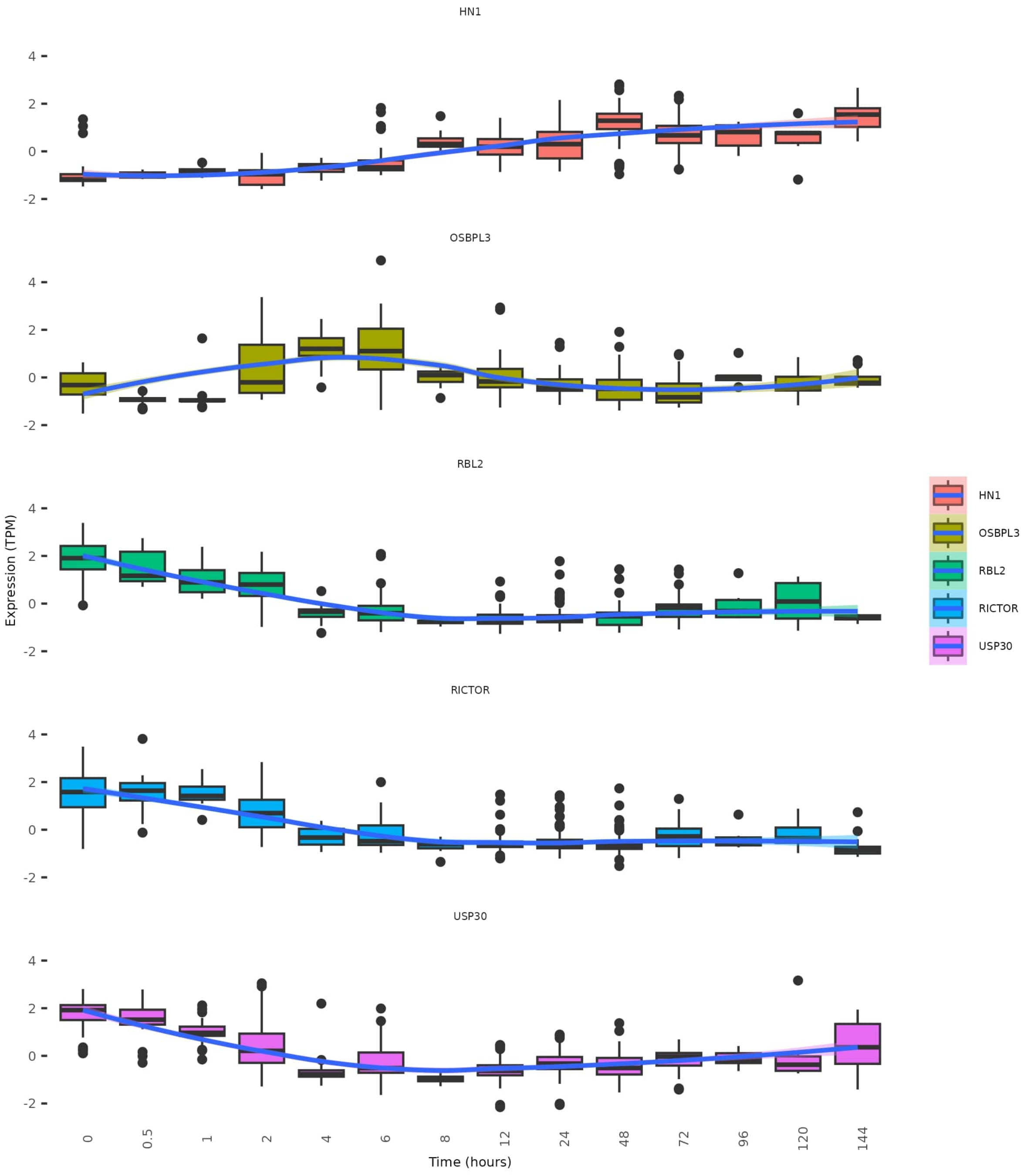

Figure S6. Expression dynamics from the T cell activation datasets of the genes that are differentially expressed in FANTOM6 CHASERR knockdown (FDR < 0.05) and are included in the T cell activation signature (FDR < 0.05) with the same direction of effect.

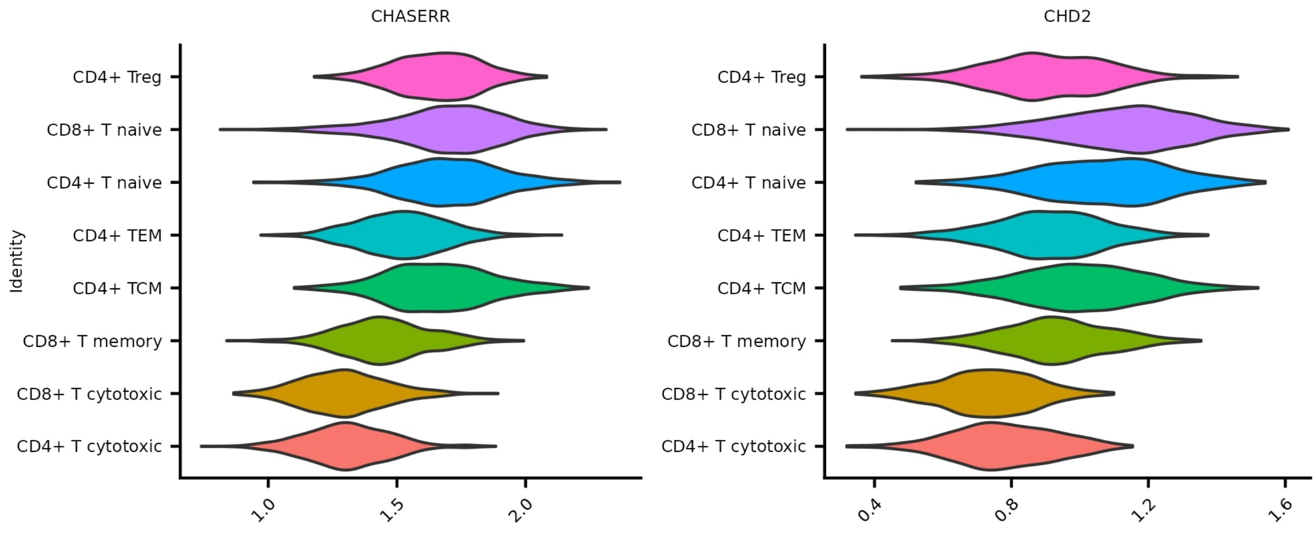

Figure S7. CHASERR and CHD2 expression in AIDA dataset metacells.

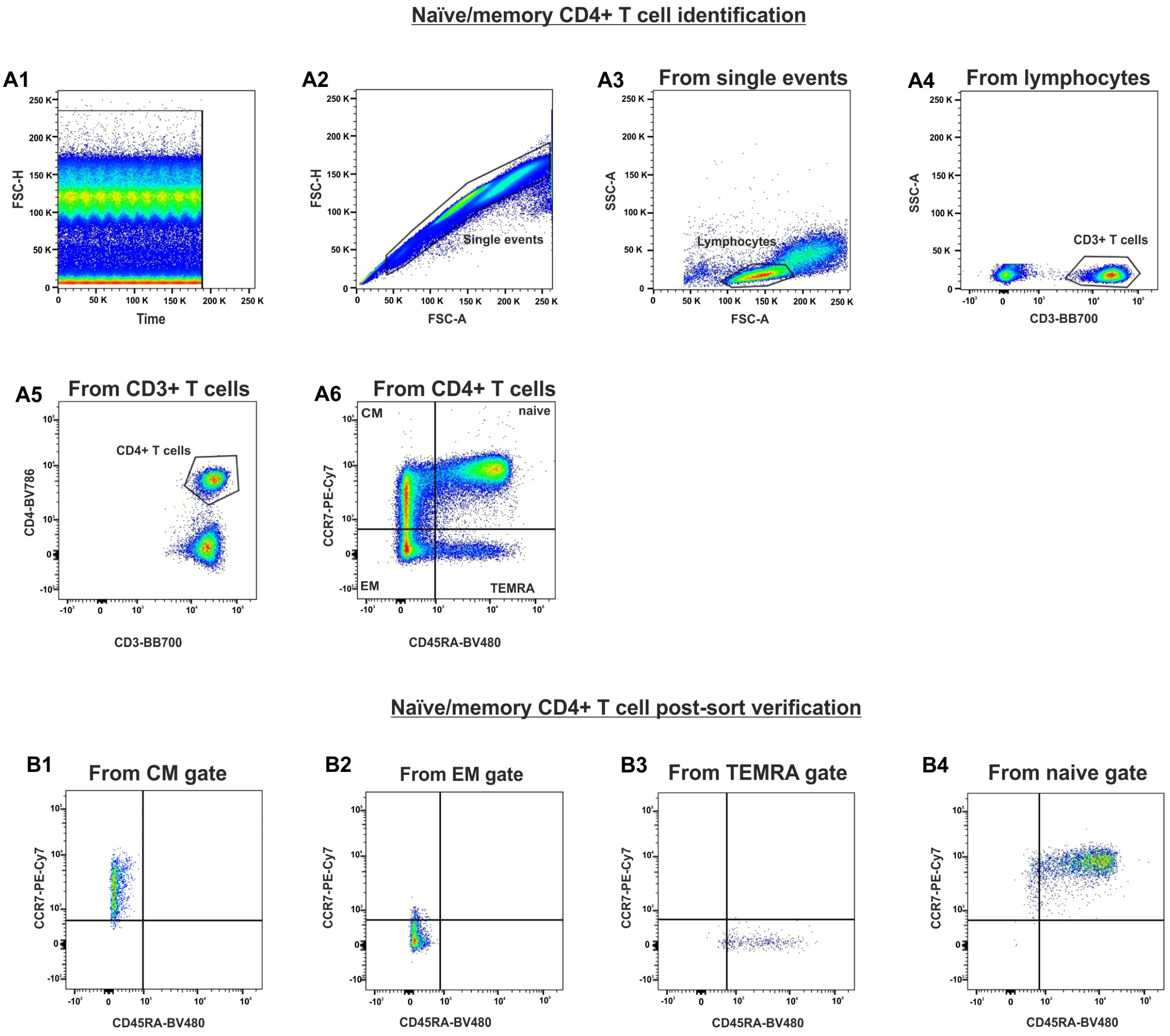

Figure S8. **(A)** Gating strategy applied to identify naïve/memory CD4+ T cell subsets from PBMC. A1 – dot plot Time/FSC-H was used to exclude any possible fluidic perturbations due to pressure jumps; A2 – dot plot FSC-A/FSC-H was used to isolate single events; A3 – dot plot FSC-A/SSC-A was used to isolate lymphocytes from monocytes; A4 – dot plot CD3-BB700/SSC was used to identify CD3+ T cells; A5 – dot plot CD3-BB700/CD4-BV786 was used to identify CD4+ T cells; A6 – dot plot CD45RA-BV480/CCR7-PE-Cy7 was used to identify naïve CD4+ T cells as CD45RA+CCR7+ cells, central memory (CM) CD4+ T cells as CD45RA-CCR7+ cells, effector memory (EM) CD4+ T cells as CD45RA-CCR7- cells; terminally differentiated effector memory (TEMRA) CD4+ T cells as CD45RA+CCR7- cells. **(B)** Verification data after sorting naïve/memory CD4+ T cell subsets using quadrant gates on CD45RA-BV480/CCR7-PE-Cy7 dot plot. B1 – cells sorted from CM CD45RA-CCR7+ gate; B2 – cells sorted from EM CD45RA-CCR7- gate; B3 – cells sorted from TEMRA CD45RA+CCR7- gate; B4 – cells sorted from naïve CD45RA+CCR7+ gate.

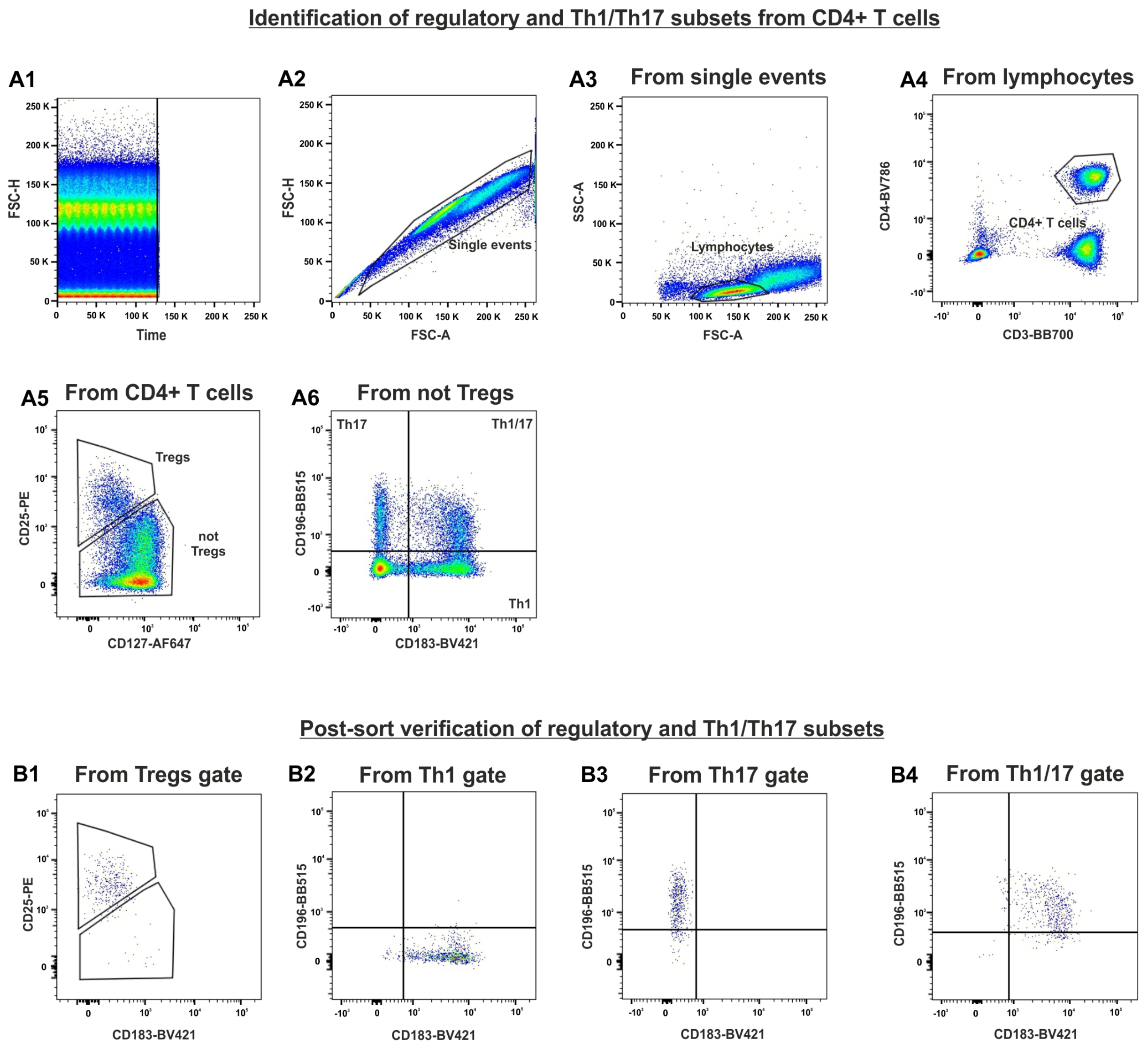

Figure S9. **(A)** Gating strategy applied to identify regulatory T cells and Th1/Th17 subsets from PBMC. A1 – dot plot Time/FSC-H was used to exclude any possible fluidic perturbations due to pressure jumps; A2 – dot plot FSC-A/FSC-H was used to isolate single events; A3 – dot plot FSC-A/SSC-A was used to isolate lymphocytes from monocytes; A4 – dot plot CD3-BB700/CD4-BV786 was used to identify CD3+CD4 Th cells; A5- dot plot CD127-AF647/CD25-PE was used to isolate regulatory T cells (Tregs) as CD127 -/low /CD25 bright and "not" Tregs as CD127+CD25-/low cells; A5 – dot plot CD183- BV421/CD196-BB515 was used to identify Th1 cells as CD183+CD196- cells, Th17 as CD183-CD196+ cells, and Th1/17 cells as CD183+CD196+ cells; **(B)** Verification data after sorting Tregs and Th1/Th17 CD4+ T cell subsets. B1 - dot plot CD127-AF647/CD25-PE used to identify Tregs as CD127 -/low /CD25 bright was further used to check the purity of Tregs sorting; B2-B4 – dot plot CD183-BV421/CD196-BB515 used to isolate of Th subsets was further used to assess the sort purity of Th1 (B2), Th17 (B3) and Th1/17 (B4) cells;

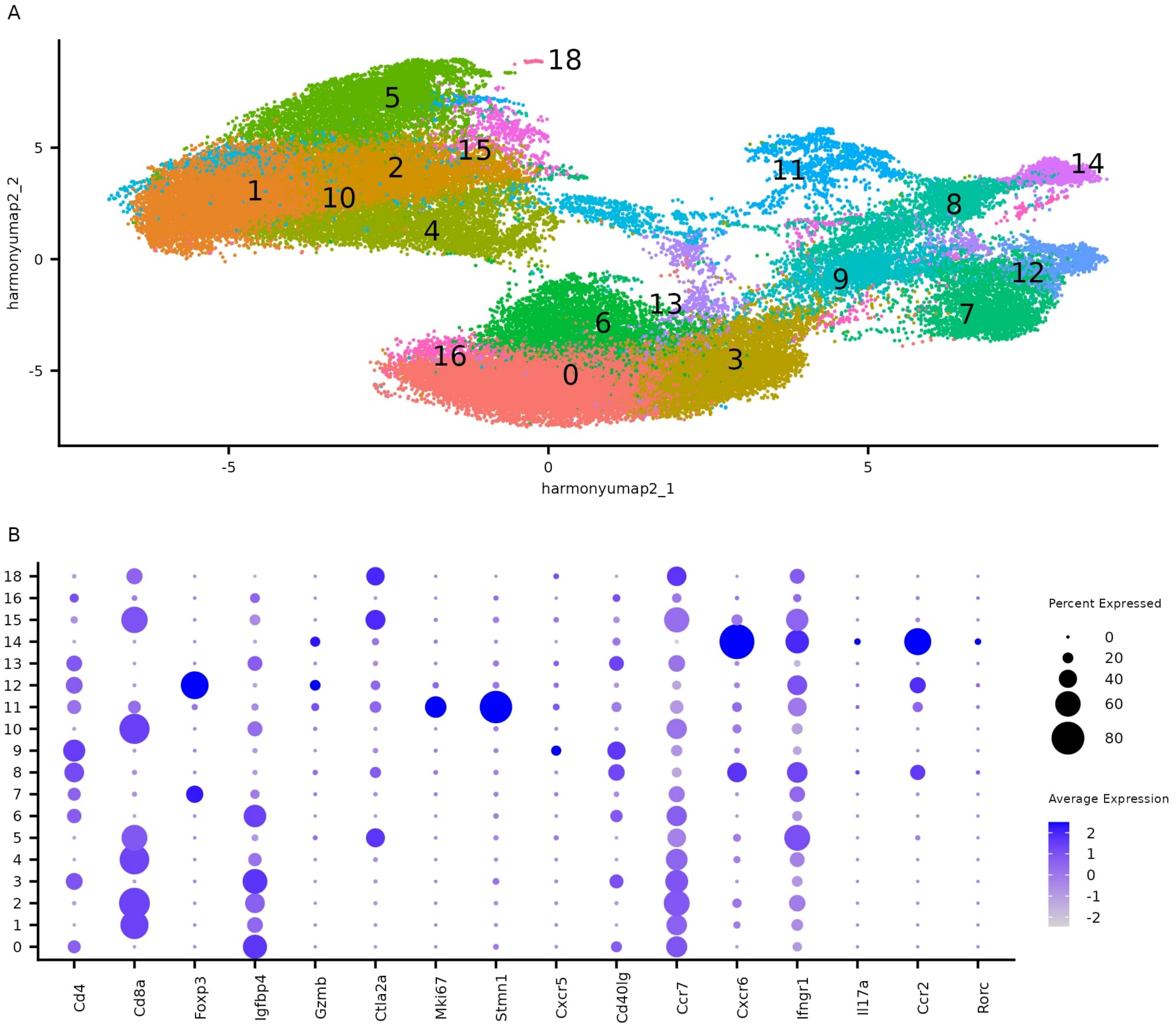

Figure S10. Cyclosporine dataset cell type annotation. **A** Cyclosporine dataset gene expression UMAP. **B** Expression of marker genes for cell annotation in the cyclosporine dataset.
